# Immobilization and Evaluation of Penicillin G Acylase on Hydroxy and Aldehyde Functionalized Magnetic α-Fe_2_O_3_/Fe_3_O_4_ Heterostructure Nanosheets

**DOI:** 10.3389/fbioe.2021.812403

**Published:** 2022-01-28

**Authors:** Yun Ni, Zhixiang Lv, Zhou Wang, Shouyu Kang, Dawei He, Ruijiang Liu

**Affiliations:** ^1^ School of Pharmacy, Jiangsu University, Zhenjiang, China; ^2^ The People’s Hospital of Danyang, Affiliated Danyang Hospital of Nantong University, Zhenjiang, China; ^3^ College of Vanadium and Titanium, Panzhihua University, Panzhihua, China; ^4^ Affiliated Kunshan Hospital, Jiangsu University, Suzhou, China

**Keywords:** magnetic α-Fe_2_O_3_/Fe_3_O_4_ heterogeneous nanosheets, immobilization, penicillin G acylase, hydrothermal calcination process, reusability

## Abstract

Magnetic α-Fe_2_O_3_/Fe_3_O_4_ heterostructure nanosheets were fabricated via hydrothermal calcination. The activity of penicillin G acylase (PGA), which was covalently immobilized onto silica-decorated heterostructure nanosheets, achieved the highest activity of 387.03 IU/g after 18 h of incubation with 0.1 ml of PGA. In contrast, the activity of free PGA reached the highest level when the temperature was 45°C with a pH of 8.0. However, the activity of free PGA changed more dramatically than immobilized PGA as the relative conditions changed. Moreover, the Michaelis–Menten constant (K_m_) and reusability of immobilized PGA were also explored. The results showed that free PGA K_m_ and maximum rate (V_max_) were 0.0274 M and 1.167 μl/min, respectively. K_m_ and V_max_ values of immobilized PGA were 0.1082 M and 1.294 μl/min, respectively. After 12 cycles of repetitive use, immobilized PGA remained approximately 66% of its initial activity, indicating that the PGA immobilized onto the heterostructure nanosheets showed better stability and reusability than free PGA.

## Introduction

Currently, magnetic materials are attracting great attention from researchers due to their promising applications in various fields, such as biotechnology (Alizadeh and Salimi, 2021), catalysis (Liu et al., 2021a), electrochemistry (Wang et al., 2021), and biosensors (Dalkıran et al., 2019). Iron (II, III) oxide (Fe_3_O_4_) nanocomposites have many applications in the enzymatic immobilization field (Li et al., 2019), as they contain the Fe^3+^ (Liu et al., 2016) and Fe^2+^ antispinel structures. Moreover, Fe_3_O_4_ nanocomposites have structural stability (Pakapongpan and Poo-arporn, 2017), light resistance, good biocompatibility, and good magnetic responsiveness. However, the magnetism of Fe_3_O_4_ produces diffusion restrictions (Liu et al., 2021b). Thus, magnetic α-iron (III) oxide (Fe_2_O_3_)/Fe_3_O_4_ heterogeneous nanomaterials (Zhuang et al., 2015) have been developed to overcome this drawback. The magnetic α-Fe_2_O_3_/Fe_3_O_4_ heterogeneous nanomaterials immobilized with enzymes (Ansari and Husain, 2012) can be separated by applying an external magnetic field, with the advantages of low toxicity, biocompatibility, simple operation, biodegradation, and effectively reduced capital investment. However, they are also accompanied by the disadvantages of low chemical stability, high concentration tendency, and low immobilized enzyme volume (Angelakeris, 2017). Hence, it is of great significance to combine inorganic or organic materials into magnetic nanocomposites (Pakapongpan and Poo-arporn, 2017) to improve material stability, delay oxidation, and enable better application of immobilized enzymes (Senapati et al., 2018). In this study, the stability of magnetic nanocomposites in an acidic environment was improved by coating them with silicon dioxide (SiO_2_). The specific surface area of the nanomaterials was significantly increased, and the abundant surface groups were suitable for the modification and transformation of nanomaterials, which was of great significance for enzymatic immobilization.

Immobilization techniques for modifying various enzymes are affected by many factors, including the carrier used for the immobilization, properties of the enzyme, solvent type, immobilization process conditions, and reaction medium (Sirisha et al., 2016). Therefore, the enzymatic immobilization approaches should be appropriately selected, according to the immobilized object characteristics and the application direction of the immobilization technology (Zhou and Hartmann, 2013). With the rapid development of science and technology, various methods of enzymatic immobilization have been developed, such as the adsorption approach (Fernandez-Lorente et al., 2020), embedding process (Nadar et al., 2020), binding process (Farid et al., 2020), and crosslinking approach (Tanabe et al., 2004). Among these, the crosslinking approach was chosen in the present study owing to the stability between the substrate and the enzyme. Commonly used crosslinking agents include dicarboxylic acid, glutaraldehyde (GA) (Rajesh et al., 2018), dibutyltin dilaurate, and dimethyl adipic imide.

Penicillin G acylase (PGA) is a critical catalyst in the biological field (Chen et al., 2018). It can hydrolyze benzylpenicillin (penicillin G) to produce side chain-free penicillin, which can be applied to the synthesis of β-lactam antibiotics in the industry (Xue et al., 2015; Xue et al., 2016). These antibiotics play a critical role in our daily lives, and they are often used to treat diseases caused by various microorganisms, ranging from viruses to bacteria (Yu et al., 2019). As a hydrolytic enzyme, PGA has mild conditions of reaction, high sensitivity, high activity, and good substrate selectivity (Liu et al., 2018). However, when free PGA is used directly, it is easily affected by pH, temperature, and other environmental conditions. In addition, it was shown to be challenging to separate free PGA from the reaction system and purify 6-aminopenicillanic acid (6-APA), a compound used for the synthesis of semisynthetic penicillin (Wang et al., 2018), resulting in product contamination and recycling difficulties. The above reasons limit the application of free PGA in the industry. Therefore, PGA immobilization has been developed to solve these problems to a certain extent (Liu et al., 2014; Yang et al., 2014).

**FIGURE 1:**
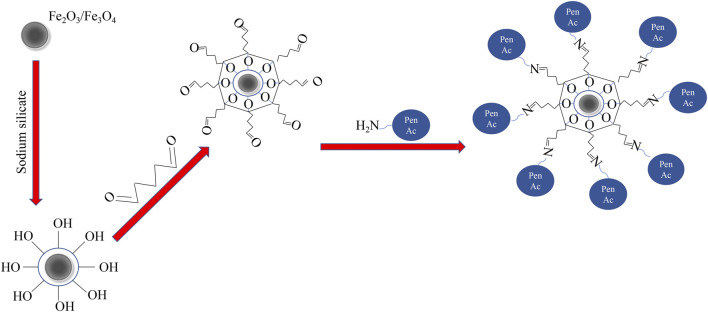
The process of penicillin G acylase (PGA) immobilized onto α-Fe_2_O_3_/Fe_3_O_4_ heterogeneous nanosheets.

In the present study, magnetic α-Fe_2_O_3_/Fe_3_O_4_ heterogeneous nanosheets were successfully fabricated via hydrothermal calcination. The magnetic nanomaterials were first coated with a silicon layer and then functionalized with glutaraldehyde (GA). PGA was successfully immobilized onto functionalized heterogeneous nanosheets, and the properties of immobilized PGA were evaluated. The immobilization process is shown in Figure 1.

## Experimental Details

### Fabrication and Characterization of α-Fe_2_O_3_/Fe_3_O_4_ Heterogeneous Nanosheets

Briefly, 0.541 g of FeCl_3_·6H_2_O and 0.094 g of NaH_2_PO_4_·2H_2_O were completely dissolved in 80 ml of distilled water by stirring with a magnetic stirrer and hydrothermally heating at 220°C for 24 h. The obtained suspension was rinsed with absolute alcohol and distilled water several times to remove impurities and then centrifuged six times. After removing the supernatant, the obtained intermedium solution was placed in a vacuum drying oven for 12 h to obtain the magnetic α-Fe_2_O_3_ nanosheets. Finally, 0.1 g of α-Fe_2_O_3_ nanosheets were uniformly mixed with 0.4 g of C_6_H_12_O_6_·H_2_O at a heating velocity of 3°C/min, and calcined at 600°C for 4 h (Liu et al., 2020). Magnetic α-Fe_2_O_3_/Fe_3_O_4_ heterogeneous nanosheets were obtained after grinding.

Scanning electron microscopy (SEM) and transmission electron microscopy (TEM) were used to evaluate the morphology of the magnetic α-Fe_2_O_3_/Fe_3_O_4_ heterogeneous nanosheets. Phase identification of the nanomaterials was performed by x-ray diffraction (XRD), and a vibrating sample magnetometer (VSM) was used to measure the magnetic properties of the products.

### Preparation of α-Fe_2_O_3_/Fe_3_O_4_@SiO_2_-CHO Nanocomposites and Penicillin G Acylase Immobilization

First, 1.0 g of α-Fe_2_O_3_/Fe_3_O_4_ heterogeneous nanosheets mixed with 200 ml of distilled water was transferred into a round bottom flask with a magnetic stirring bar and heated at 80°C for 3 h. Then 10 ml of 1.0 M Na_2_SiO_3_ was slowly added to the resulting suspension with rapid stirring. Simultaneously, a small amount of 2.0 M HCl was added to the solution to maintain pH 6.0. The magnetic α-Fe_2_O_3_/Fe_3_O_4_@SiO_2_ nanomaterials were successfully obtained after several rounds of washing and centrifugation, and finally drying. Next, 0.1 g of magnetic α-Fe_2_O_3_/Fe_3_O_4_@SiO_2_ nanomaterials were placed in a mini centrifuge tube, and 0.2 ml of 25% glutaraldehyde (Sinopharm Chemical Reagent Co., Ltd., Shanghai, China) with 1 ml of 0.05 M phosphate-buffered saline (PBS, pH 7.0) was added to the tube and stirred for 2 h. After this period, the solution was centrifuged, and the supernatant was removed. Magnetic α-Fe_2_O_3_/Fe_3_O_4_@SiO_2_-CHO nanocomposites were successfully fabricated after rinsing with 1 ml of 1.0 M NaCl.

Then, 4.6 ml of diluted PGA solution, prepared with 0.1 ml penicillin and 4.5 ml PBS (pH 8.0), was added to 0.1 g of magnetic α-Fe_2_O_3_/Fe_3_O_4_@SiO_2_-CHO nanocomposites. The solution was uniformly mixed following a shaking time of 18 h. After centrifugation, the content of enzymatic protein in the upper liquid was stained with Coomassie blue G250 (Sinopharm Chemical Reagent Co., Ltd., Shanghai, China) for its quantification.

### Thermostability and pH Effect on Penicillin G Acylase Enzymatic Activity

First, 2 ml of PBS (pH 8.0) or 4 μl of PGA were mixed with 0.1 g of magnetic α-Fe_2_O_3_/Fe_3_O_4_@SiO_2_-CHO nanocomposites immobilized with PGA. Five milliliters of 4% penicillin K solution (Aladdin, Shanghai, China) was added to each mixture and incubated for 5 min at room temperature After this time, the mixture was centrifuged, and 1 ml of the supernatant was added to 4 ml of deionized water to compose a 0.5 ml of mixture, and later combined with 3.5 ml of paradimethylaminobenzaldehyde. The UV-vis absorbance of this final solution was measured at 415 nm after an incubation time of 5 min.

For measuring the pH effects, 2 ml of PBS with various pH values (ranging from pH 6.0 to 9.0) was added to 4 μl of free PGA. Simultaneously, 0.1 g of α-Fe_2_O_3_/Fe_3_O_4_ nanosheets immobilized with PGA, was mixed with 2 ml of PBS at various pH values (6.0–9.0). Enzymatic activity was monitored based on the steps mentioned above.

To evaluate the thermostability of the composites, free and immobilized PGAs were mixed with the PBS solution with the pH value that resulted in higher enzymatic activity, and heated at various temperatures (ranging from 20°C to 60°C) for 5 min. Enzymatic activities were monitored based on the previous experimental steps.

Twenty mini centrifuge tubes were prepared, and 0.5 ml of free PGA and 2 ml of PBS were added to each tube. The tubes were divided into four groups and heated at 30°C, 40°C, 50°C, or 60°C. One sample from each group was heated during five different periods (2–10 h). Subsequently, 5 ml of 4% penicillin K solution at room temperature was added to each one of the 20 tubes. In another set of 20 mini centrifuge tubes, 0.1 g of nanosheets immobilized with PGA was added to 2 ml of PBS only. Enzymatic activity was monitored based on the previously mentioned experimental steps.

### Immobilized Penicillin G Acylase Kinetics and Reusable Property

To study the enzymatic kinetics of immobilized PGA, various concentrations of penicillin K solution (0.01, 0.0125, 0.017, 0.025, and 0.05 mM) at 37°C were used to determine the initial hydrolysis rates of PGA, using the Lineweaver-Burk plot.

The immobilized PGA property was evaluated by adding 5 ml of 4% penicillin K solution to a mixture of 0.1 g of the nanomaterials immobilized with PGA plus 2 ml of PBS after 5 min. The UV-vis absorbance of the supernatant at 415 nm was measured after centrifugation. The procedures mentioned above were repeated after various rinsing steps with PBS, until the activity of the immobilized PGA was reduced to a certain extent.

## Results and Discussion

### Characterization of α-Fe_2_O_3_/Fe_3_O_4_ Nanosheets and α-Fe_2_O_3_/Fe_3_O_4_@SiO_2_ Nanocomposites

**FIGURE 2:**
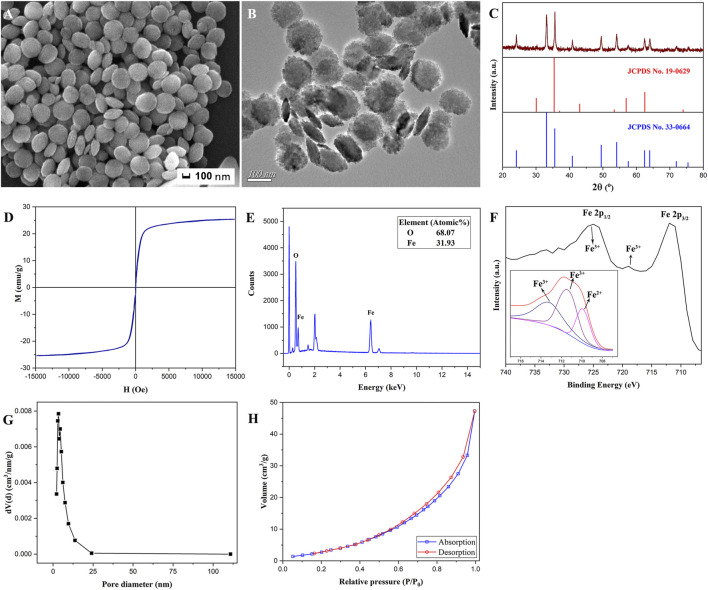
The scanning electron microscopy (SEM) morphology **(A)**, transmission electron microscopy (TEM) image **(B)**, x-ray diffraction (XRD) pattern **(C)**, hysteresis loops **(D)** EDS spectrum **(E)**, x-ray photoelectron spectroscopy (XPS) spectrum **(F)**, the chart of pore diameter distributions **(G)**, and N_2_ adsorption and desorption diagram **(H)** of magnetic α-Fe_2_O_3_/Fe_3_O_4_ heterogeneous nanosheets.

The SEM morphology (Figure 2A) and TEM image (Figure 2B) of the prepared heterogeneous nanosheets calcined at 600°C for 4 h showed that the product morphology remained as nanosheets and did not change after calcination. It can be seen that the magnetic α-Fe_2_O_3_/Fe_3_O_4_ nanosheets exhibited slight aggregation due to the magnetism of the heterogeneous nanosheets. However, they are generally well dispersed. The average diameter and thickness, measured with the Nano Measurer, reached approximately 240 and 40 nm, respectively. The x-ray diffraction (XRD) pattern of the magnetic α-Fe_2_O_3_/Fe_3_O_4_ heterogeneous nanosheets is depicted in Figure 2C. The characteristic peaks at 24.1°, 33.1°, 35.6°, 40.8°, 49.4°, 54.0°, 62.4°, and 63.9° (Liu et al., 2020) were consistent with those of the standard card of Fe_2_O_3_ (JCPDS No. 33-0664), which represent 012, 104, 110, 113, 024, 116, 214, and 300 crystal faces, respectively, indicating the formation of hematite (Mehdizadeh et al., 2020). However, the ratio of the sample peak intensities at 33° and 35.7° was significantly lower than that of the standard Fe_2_O_3_ card. This phenomenon could be attributed to the existence of Fe_3_O_4_, as the peak at 35.7° exhibited a higher intensity. Based on the standard card of Fe_3_O_4_ (JCPDS No. 19-0629), the peak intensity of Fe_3_O_4_ at 35.7° was higher than the peak intensity at 33°. In contrast, Fe_2_O_3_ exhibited a higher peak intensity at 33° than at 35.7°. Hence, this phenomenon suggests the successful formation of Fe_2_O_3_/Fe_3_O_4_ heterogeneous nanomaterials. Figure 2D shows that the magnetic saturation strength of the prepared heterogeneous nanosheets was 25.1 emu/g, which was slower than the previously prepared Fe_2_O_3_/Fe_3_O_4_ nanoparticles and Fe_2_O_3_/Fe_3_O_4_ nanotubes. However, the magnetic saturation strength of the Fe_2_O_3_/Fe_3_O_4_ nanosheets was higher than that of the Fe_2_O_3_/Fe_3_O_4_ nanorods. Therefore, we could apply the external magnetic field method to attract immobilized materials for separation and reduce the aggregation of magnetic nanomaterials.

The element types and contents of the fabricated nanomaterials are shown in Figure 2E. The proportion of Fe to O was 0.47, according to the data in the figure, meaning that divalent iron and ferrous iron (Liu et al., 2020) might exist. The x-ray photoelectron spectroscopy (XPS) of the Fe_2_O_3_/Fe_3_O_4_ heterostructure nanosheets is shown in Figure 2F. As investigated in previous studies, the characteristic peaks at around 711 and 724 eV represent the Fe 2p1/2 and Fe 2p3/2 curves, the positions of which were relevant to the ionic states of Fe. The peak at approximately 719 eV corresponds to the satellite peak of Fe^3+^. With the increase in iron oxidation in the compound, the characteristic peaks all moved toward high binding energy. The Gauss–Lorenz method was adopted to fit the Fe 2p3/2 curve. Peaks of high binding energy at approximately 711 and 713 eV were attributed to Fe^3+^, while the peak at approximately 709 eV was attributed to Fe^2+^, which indicated the existence of Fe_3_O_4_. The BET spectra of magnetic α-Fe_2_O_3_/Fe_3_O_4_ heterogeneous nanosheets are displayed in Figures 2G,H. The average pore diameters were focused on 3–4 nm, and the specific surface area of the prepared heterostructure nanosheets was approximately 14.82 m^2^/g.

**FIGURE 3:**
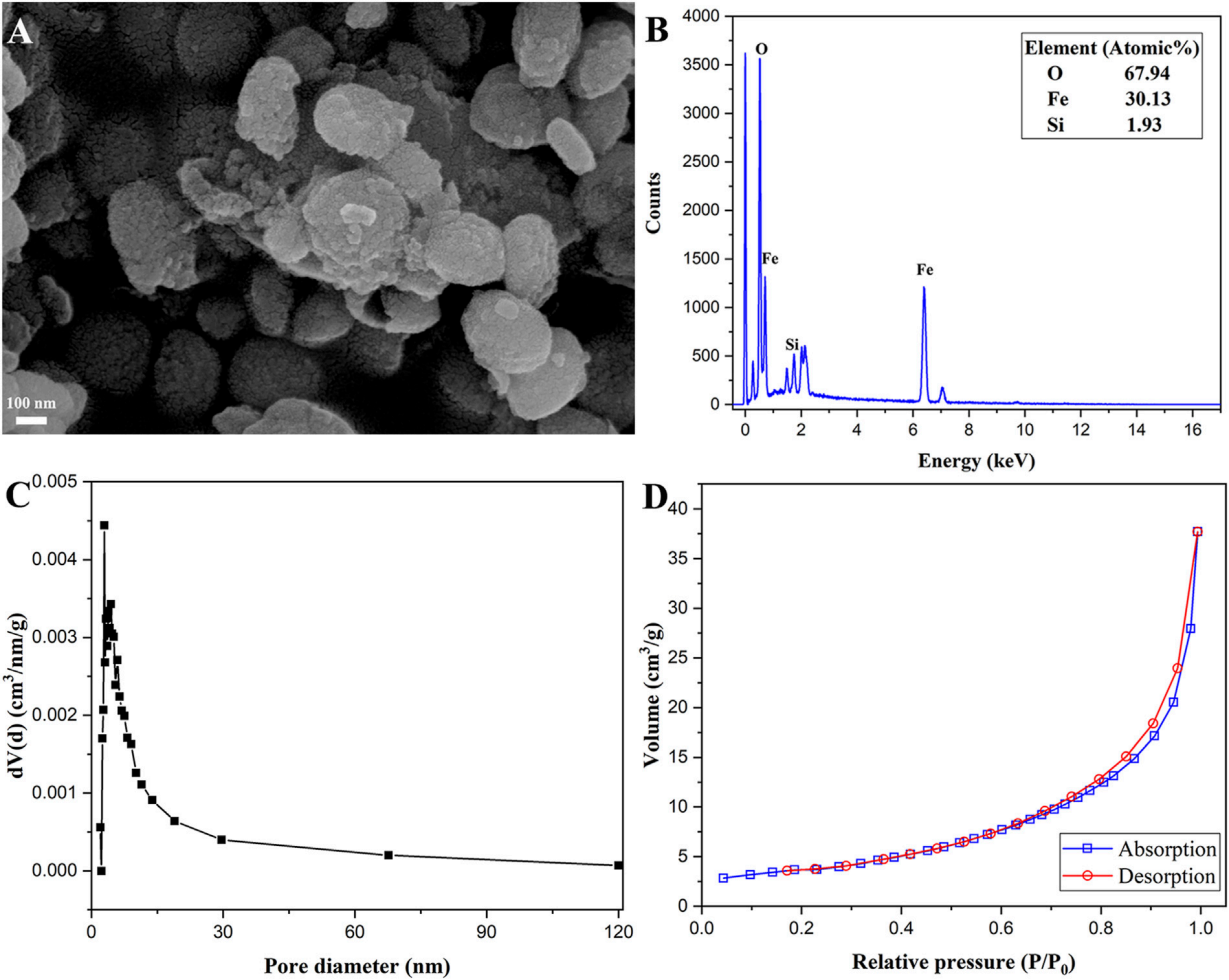
SEM morphology **(A)**, EDS spectrum **(B)**, the chart of pore diameter distributions **(C)**, and N_2_ adsorption and desorption diagram **(D)** of α-Fe_2_O_3_/Fe_3_O_4_@SiO_2_ nanocomposites.

The SEM morphology of the magnetic α-Fe_2_O_3_/Fe_3_O_4_@SiO_2_ nanocomposites is shown in Figure 3A. The surface of the magnetic α-Fe_2_O_3_/Fe_3_O_4_@SiO_2_ nanocomposites was rough, which could be attributed to the SiO_2_ coating. The magnetic α-Fe_2_O_3_/Fe_3_O_4_@SiO_2_ nanocomposites also showed slight aggregation, proving the existence of magnetism in the prepared nanocomposites. The EDS spectrum of Fe_2_O_3_/Fe_3_O_4_@SiO_2_ is shown in Figure 3B, revealing that the elemental composition of the prepared products included Si, O, and Fe. As observed in Figures 3C,D, the specific surface area of the silica-decorated heterostructure nanosheets was approximately 12.65 m^2^/g. The size of the specific surface area was mostly related to the pore structure of the material. The pore diameter of the nanocomposites was approximately 3 nm. The specific surface area and mesoporous structure increased the possibility of enzymatic immobilization onto magnetic nanomaterials.

### Molecular Docking Analyses

**FIGURE 4:**
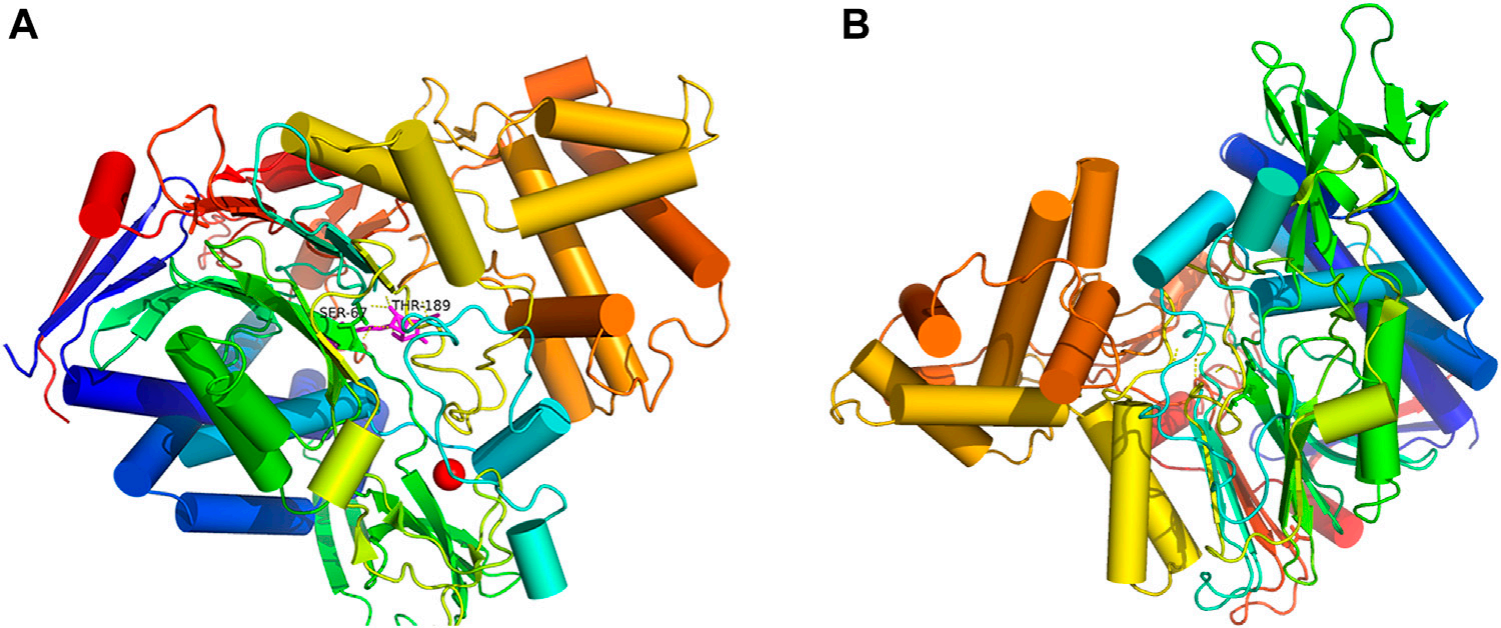
Orientation confirmation of PGA **(A)**, and molecular docking mode of penicillin K and PGA **(B)**.

The automatic docking enzyme program (Zhang et al., 2020) was implemented to generate the docking between PGA and penicillin K, and the results are shown in Figure 4. The location of the active sites might influence the activity of the enzyme. This property enhanced the binding affinity of PGA to the substrate. The presence of Fe_2_O_3_ weakened the diffusion limitation caused by Fe_3_O_4_ (Esmaeili et al., 2011). However, these processes could not be performed using bare Fe_2_O_3_/Fe_3_O_4_ heterogeneous nanosheets. Therefore, it was necessary to create an appropriate interface for the active site. SiO_2_ can provide a large amount of silica hydroxyl groups, which can be used as a coating on the surface of heterogeneous nanosheets and immobilize the carrier of PGA (Pan et al., 2019; Chen et al., 2020). In addition, a suitable immobilization method was used. Glutaraldehyde (GA) is a powerful crosslinking agent that reacts with the free amino group in PGA and does not damage PGA after crosslinking with its active site (Liu et al., 2020). The kinetics model was established by molecular docking to study the mechanism of enzyme immobilization and find the best binding pattern between the two molecules, which was of great value for investigating the immobilization conditions.

### Optimization of Penicillin G Acylase Immobilization Conditions

**FIGURE 5:**
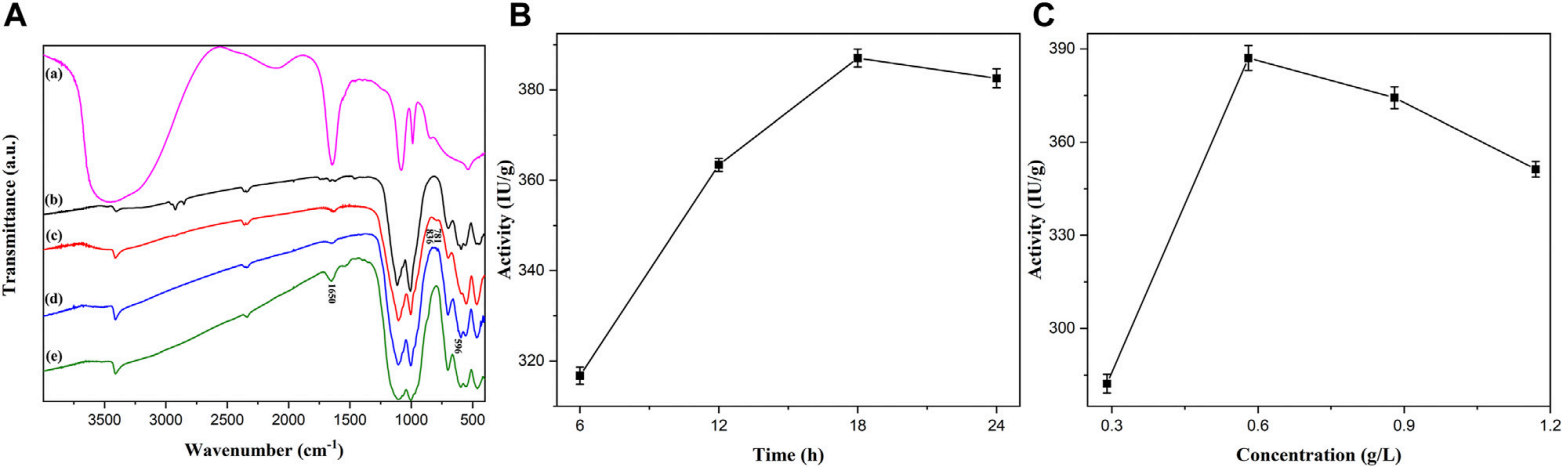
The Fourier transform infrared (FTIR) spectra **(A)** of the PGA immobilization process and the influences of time **(B)** and concentration **(C)** on the activity of PGA.

Overall, Figure 5A shows the Fourier Transform infrared spectroscopy (FTIR) of PGA, Fe_2_O_3_/Fe_3_O_4_ nanosheets, Fe_2_O_3_/Fe_3_O_4_@SiO_2_, α- Fe_2_O_3_/Fe_3_O_4_@SiO_2_-CHO, and Fe_2_O_3_/Fe_3_O_4_@SiO_2_-CH=N-PGA. Figure 5A (a) shows the absorption peaks of PGA. As illustrated in Figure 5A (b), the stretching vibration of the Fe–O bond from magnetic Fe_2_O_3_/Fe_3_O_4_ heterogeneous nanosheets was significant to the absorption peak at 550 cm^−1^. The peaks at around 781 and 836 cm^−1^ in Figure 5A (c) successfully evidenced the SiO_2_ coating of Fe_2_O_3_/Fe_3_O_4_ nanomaterials, due to the stretching vibration of the Si–O bond. Similar to Figure 5A (c), the stronger peak caused by the modification of GA appeared at approximately 596 cm^−1^ in Figure 5A (d). The enhanced absorption peak at 1,650 cm^−1^ in Figure 5A (e) corresponds to the characteristic peak of PGA, which is caused by the stretching vibration of the C=N bond. FTIR spectrum analysis showed that PGA was successfully immobilized onto the silica-decorated heterostructure nanosheets. Thenceforth, to obtain the optimum time for PGA immobilization, 2 ml of PBS was shaken with immobilized PGA for various periods (6–24 h) at a frequency of 115 rpm. After each incubation time, 5 ml of the 4% penicillin K was added to the PGA solution, followed by another 10 min of reaction. The enzymatic activity of the centrifuged supernatant is shown in Figure 5B, which shows that the activity trend first increased and then dropped with the extension of immobilization time. The enzymatic activity reached the highest level of 387.03 IU/g at 18 h. The reason for the decreased activity was that the immobilized PGA was inactivated with extended immobilization time. The influence of various concentrations on immobilized PGA is represented in Figure 5C. First, 2 ml of PBS was prepared with different amounts of free PGA (0.05, 0.1, 0.15, and 0.2 ml). The following experimental procedures for free PGA were similar to those of the immobilized PGA mentioned above. The activity initially exhibited an upward trend and then decreased. When the concentration was 0.58 g/L, the enzymatic activity reached the highest level. The increasing trend might be due to the magnetic nanomaterials that were not completely connected with the PGA. As the concentration increased, excess PGA was not covalently bonded with the nanomaterials but was absorbed on the nanomaterials, which resulted in a smaller specific surface area and masking of the active spots. The decreasing trend after 18 h was caused by less contact between the immobilized enzyme and its substrate.

### Evaluation of Immobilized and Free Penicillin G Acylase Kinetics and Reproducibility

**FIGURE 6:**
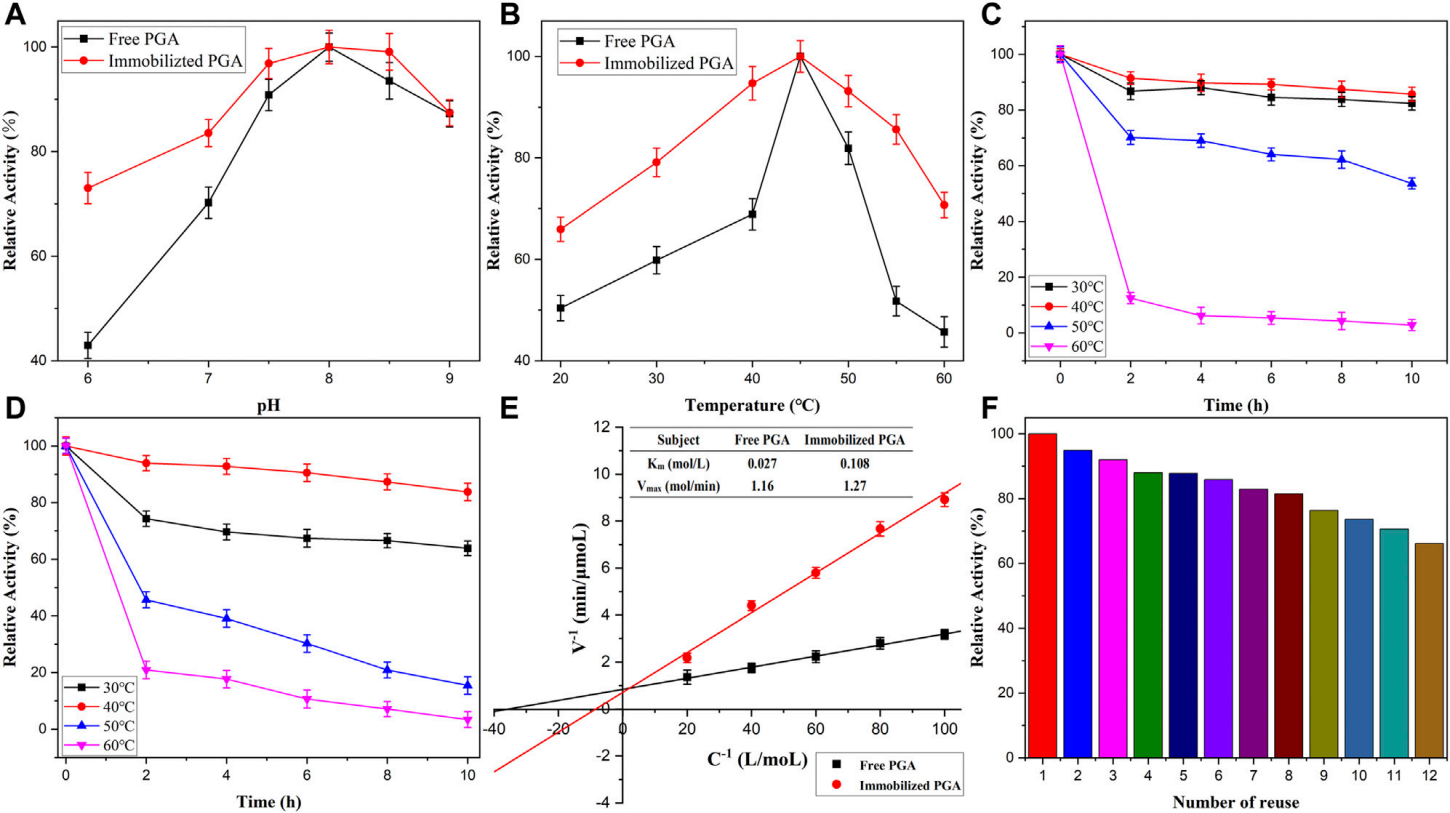
The pH value **(A)**, temperatures **(B)**, and thermostabilities on free **(C)** and immobilized PGA **(D)**, the Lineweaver-Burk diagram **(E)** and the reproductivity of immobilized PGA **(F)**.

The influence of pH and thermostability was investigated to monitor the highest activity of immobilized and free PGA. The results in Figure 6A show that the trend of the enzyme activity increased first and then dropped with the pH increment, reaching the highest level at pH 8.0. Therefore, the activities of free PGA at 20°C–60°C were determined at pH 8.0 after 10 min of reaction. As shown in Figure 6B, the activity increased first and then dropped dramatically with the increment in the temperature of the reaction, reaching the maximum activity at 45°C. As observed from the graph, free PGA activity varied more dramatically than the immobilized PGA, which suggested that the immobilized PGA exhibited greater thermal stability than free PGA. Above all, the nanomaterials improved the structure of PGA, preventing conformational changes and degeneration in extreme environments. The thermostability was analyzed based on previous experiments, as shown in Figures 6C,D, respectively. The immobilized and free PGAs were cultured in PBS (pH 8.0) at 30°C, 40°C, 50°C, and 60°C for different intervals (2–10 h). When the heating time and the reaction temperature increased, the relative activity of both immobilized and free PGA decreased to a certain degree. As observed clearly in the figure, the activity dropped intensely at 50°C and 60°C. This phenomenon may be attributed to the destruction of the activity center. Immobilized PGA exhibited higher activity than free PGA at 30°C and 40°C. This could be because the immobilized carrier decreased the conformational change of the active center of the PGA. The conclusion drawn from these analyses reveals that PGA immobilized onto magnetic Fe_2_O_3_/Fe_3_O_4_ heterogeneous nanomaterials may be conducive to the application of enzymes.

Different concentrations of penicillin K acting as the substrate reacted with free and immobilized PGA during different periods. The corresponding initial velocities were measured by linear regression analysis after measuring the amount of 6-aminopenicillanic acid (6-APA).

The Lineweaver-Burk diagram was constructed by linear regression analysis, as shown in Figure 6E. The K_m_ value (0.108 mM) of the immobilized PGA was approximately four times the K_m_ value (0.027 mM) of free PGA. This could be attributed to the formation of a diffusion layer around the immobilized PGA, which had a stereoscopic effect on the substrate. Therefore, this may reduce the binding of the PGA active site with its substrate and lessen the affinity between the immobilized PGA and the substrate.

The regenerative performance of PGA can determine its practical value in industrial applications. The reusability of immobilized PGA is clearly shown in Figure 6F, and the percentage of residual activity was determined using the initial activity as the control (100%). As revealed in the picture, the activity of immobilized PGA onto nanomaterials dropped with its reuse, which was associated with the loss of some parts of the enzyme in the carrier separation. However, the activity remained around 66% after multiple cycles, which suggested that the immobilized PGA exhibited good reusability (Sheldon and Van Pelt, 2013).

## Conclusion

Magnetic α-Fe_2_O_3_/Fe_3_O_4_ heterogeneous nanosheets were fabricated via a hydrothermal calcination process (Liu et al., 2020; Hong et al., 2021). The magnetic saturation strength of the prepared nanosheets reached a maximum of 25.1 emu/g after calcination for 4 h. The average diameter and thickness of the magnetic nanosheets were approximately 240 and 40 nm, respectively. PGA was immobilized onto the modified carrier by covalent crosslinking, which effectively improved the catalytic performance of PGA. In contrast to free PGA, immobilized PGA exhibited better stability and reusability, with catalytic activity remaining at 66% of the initial activity after 12 cycles. The present study suggests that immobilizing PGA onto magnetic α-Fe_2_O_3_/Fe_3_O_4_ heterogeneous nanosheets can play a critical role in catalytic applications.

## Data Availability

The original contributions presented in the study are included in the article/Supplementary Material, further inquiries can be directed to the corresponding authors.
